# Introduction to the special issue on epigenetic regulation of chronic pain

**DOI:** 10.1097/PR9.0000000000001406

**Published:** 2026-01-20

**Authors:** Andrea G. Nackley

**Affiliations:** a Department of Anesthesiology, Center for Translational Pain Medicine, Duke University School of Medicine, Durham, NC, USA; b Department of Pharmacology and Cancer Biology, Duke University, Durham, NC, USA

## Abstract

This Special Issue features 6 articles from leaders in the field that elucidate novel epigenetic mechanisms regulating nociception, inflammation, responses to pharmacologic and integrative therapies, and pain disparities among racial/ethnic groups. Together, they highlight the expanding potential of epigenomics to inform mechanistic discovery, guide personalized pain therapeutics, and advance pain equity.

## 1. Introduction

Epigenetic mechanisms, including DNA methylation, histone modification, and noncoding RNA, provide dynamic molecular pathways through which injury, inflammation, psychological stress, and environmental exposures leave lasting biological imprints. These marks can influence transcriptional programs in neurons, immune cells, glia, adipocytes, and other tissues relevant to chronic pain. Because epigenetic changes are *reversible* and *context dependent*, they provide biological pathways that can encode the “memory” of prior insults, a concept central to persistent pain.

Importantly, a bidirectional relationship exists between epigenetics and analgesics, whereby analgesics can shape epigenetic states and epigenetic variation can influence the efficacy and safety of analgesic therapies. Thus, unpacking the pain epigenome will advance our understanding of chronic pain pathophysiology, inform current pain management approaches, and facilitate design and discovery of novel analgesic campaigns.

Preclinical studies demonstrate robust epigenetic alterations after nerve injury, inflammation, and stress, including genome-wide DNA methylation shifts, altered histone acetylation, and differential expression of microRNAs. Complementary clinical studies reveal epigenetic signatures in chronic pain conditions, suggesting potential biomarkers of susceptibility, phenotype, and treatment response.

Despite rapid progress, major challenges remain. Determining causal relationships, establishing cell-type–specific epigenetic signatures, and integrating genomic, socio-environmental, and psychosocial factors require interdisciplinary approaches and advanced analytic frameworks.

This Special Issue of PAIN Reports brings together 6 contributions that collectively advance our understanding of how epigenomic processes influence pain onset, maintenance, treatment response, and disparities. The papers are organized thematically across 3 domains: (1) epigenetic regulation of nociception and inflammation; (2) therapeutic modulation of the epigenome; and (3) epigenetics, environment, and pain disparities.

### 1.1. Epigenetic regulation of nociception, inflammation, and pain vulnerability

A central theme emerging across molecular pain research is that epigenetic states shape how tissues respond to noxious stimuli, inflammation, and stress. The 3 papers in this section approach this concept from complementary angles: dissecting DNA methylation, intracellular microRNA regulation, and extracellular vesicle–mediated signaling to illustrate how epigenetic alterations contribute to pain onset and maintenance across distinct biological contexts.

Stone and colleagues provide a uniquely controlled comparison of symptomatic and asymptomatic intervertebral discs from the same individual.^6^ Despite comparable structural degeneration, painful discs exhibited distinct DNA methylation signatures involving genes related to extracellular matrix remodeling, immunomodulation, ion channel function, and long noncoding RNAs. Enriched pathways included immune response, hormonal signaling, and musculoskeletal development. These findings demonstrate how local epigenomic states may determine whether degeneration becomes painful, highlighting the importance of tissue-specific epigenetic patterns in chronic pain vulnerability.

Extending this theme, Nackley and colleagues identify the miR-374 family as a convergent regulator of chronic primary pain across 3 human cohorts and a clinically relevant mouse model of primary pain onset.^2^ miR-374 was consistently downregulated in plasma, adipose tissue, and spinal cord during pain onset, with female-specific reductions in plasma and spinal tissue. Mechanistic studies linked adrenergic activation to suppression of miR-374 in adipocytes, and miR-374 overexpression reduced nociceptor activity. This work underscores how epigenetic mediators shape tissue-specific contributions to persistent pain.

Ajit and colleagues broaden the mechanistic landscape by demonstrating that microRNAs also act as intercellular epigenetic messengers.^1^ miRNAs such as miR-939, implicated in complex regional pain syndrome, are selectively packaged into small extracellular vesicles through EXOmotif sequences and RNA-binding proteins. These vesicles circulate between cells to modulate inflammatory signaling. Viewed alongside the miR-374 findings, this work suggests a multiscale model in which microRNAs function both within and between cells to coordinate nociceptive and inflammatory pathways.

In sum, pain vulnerability reflects the interaction of localized epigenetic alterations within tissues and broader regulatory networks involving microRNAs and intercellular communication.

### 1.2. Epigenetic mechanisms influencing response to pain therapies

The next 2 papers demonstrate that epigenetic regulation not only contributes to pain susceptibility but also influences how individuals respond to therapy and, conversely, that therapy itself can remodel the epigenome. Chidambaran and colleagues show that DNA methylation surrounding the OPRM1 A118G variant predicts acute postoperative pain, chronic postsurgical pain, and opioid-related respiratory depression in adolescents undergoing major surgery.^5^ Several 5'—C—phosphate—G—3' sites within the OPRM1 promoter and downstream region, independent of genotype, were associated with pain and safety outcomes and mapped to Repressed Polycomb chromatin states. These findings indicate that epigenetic variation at opioid receptor loci influences both analgesic efficacy and risk, reinforcing that treatment variability is rooted not only in genetics but also in epigenomic context.

Park et al. present evidence that acupuncture induces epigenomic remodeling across multiple brain regions involved in pain and affective processing.^3^ Acupuncture modulated DNA methylation of genes related to mitochondrial function, oxidative phosphorylation, inflammation, and neurogenesis as well as changes associated with improvements in neuropathic pain symptoms and related cognitive and emotional sequelae. This work illustrates how integrative therapies can normalize dysregulated epigenetic states.

In sum, these studies demonstrate how epigenetic factors shape therapeutic response, and how therapeutic interventions can restore or reprogram maladaptive epigenetic states. Further, they highlight emerging opportunities for epigenetically informed analgesic strategies.

### 1.3. Epigenetics, environment, and pain disparities

The final paper expands the epigenetic lens to include the social and structural determinants of pain, demonstrating how environmental adversity becomes biologically embedded to influence pain outcomes. Aroke et al. provide evidence that chronic stress, discrimination, and socioeconomic adversity alter DNA methylation in genes regulating stress reactivity and neuroinflammation.^4^ They highlight growing data that epigenetic age acceleration mediates relationships between social disadvantage and pain severity. Although epigenetic mechanisms help explain biological manifestation of disparities, the authors emphasize that structural inequities are the root cause, and epigenetic insights should be used to inform but not replace social and policy solutions.

## 2. Conclusion

Epigenetics is reshaping our understanding of chronic pain by revealing how environmental factors, injury, and therapeutic experiences leave enduring molecular marks that influence pain trajectory. The 6 papers in this special issue illustrate the conceptual breadth and translational promise of epigenetic science from genetic susceptibility to intra- and intercellular mechanisms, therapeutic responses, and clinical pathology. We hope this collection catalyzes interdisciplinary insight and accelerates future discovery toward interventions that prevent, reverse, or fundamentally reprogram chronic pain.

## Disclosures

The author has no conflict of interest to declare.
